# Fabrication of anti-icing/de-icing surfaces by femtosecond laser

**DOI:** 10.3389/fchem.2022.1073473

**Published:** 2022-11-24

**Authors:** Bo-Hao Tang, Qiang Wang, Xing-Chen Han, Hao Zhou, Xiao-Jing Yan, Yi Yu, Dong-Dong Han

**Affiliations:** ^1^ Changchun Institute of Optics, Fine Mechanics and Physics, Chinese Academy of Sciences, Changchun, China; ^2^ State Key Laboratory of Integrated Optoelectronics, College of Electronic Science and Engineering, Jilin University, Changchun, China; ^3^ Key Laboratory of Icing and Anti/De-icing, China Aerodynamics Research and Development Center, Mianyang Sichuan, China

**Keywords:** anti-icing surface, de-icing surface, bioinspired surface, micro-/nano-fabrication, femtosecond laser

## Abstract

In this minireview, we comprehensively reviewed recent progress on fabricating anti-icing/de-icing surfaces by femtosecond laser technologies. Typical bioinspired micro-/nano-structures fabrication strategies, superhydrophobic surfaces with anti-icing properties, and photothermal surfaces with de-icing properties are summarized. At last, we discussed challenges and prospects in anti-icing/de-icing surfaces fabricated by femtosecond laser technologies.

## 1 Introduction

Icing shows serious problems in power, energy, and communications (Pan et al., 2015; Han et al., 2020a). Traditional de-icing technologies include manual, mechanical, thermal, laser, electromagnetic, and ultrasonic field (Dhyani et al., 2022; Huang et al., 2022). Among various traditional de-icing technologies, manual de-icing technology is the most commonly used. But manual de-icing is low efficiency and high cost (Cheng et al., 2022; Patel et al., 2022). In the past decade, inspired by nature, significant progress in de-icing/anti-icing has been developed (Yi et al., 2021; Jiao et al., 2023). For example, inspired by the superhydrophobic properties of lotus leaves, researchers have successfully prepared superhydrophobic surfaces for anti-icing (Han et al., 2020b; Chen et al., 2022). Inspired by moth eyes, micro-/nano-structures convert light into thermal energy under sunlight irradiation leading to ice melting, which is energy-saving, environmentally friendliness, and low-cost (Zhao et al., 2021; Chen et al., 2022; Liu et al., 2022).

Femtosecond laser fabrication technologies have advantages in ultrashort pulse duration, ultra-high instantaneous power, ultra-fine processing structure (You et al., 2020; Zheng et al., 2020; Fu et al., 2021; Zhang et al., 2021; Jin et al., 2022). In particular, the fine micro-/nano-structures play a vital role in the aspect controlling surface wettability, such as de-icing, anti-icing, superhydrophobic, superoleophobic, and slippery surface (Yong et al., 2017; Yong J. et al., 2019; Feng and Yong, 2020; Yong et al., 2020; Yong et al., 2022a). Compared with other micro-/nano-fabrication technologies, femtosecond laser shows distinguish advantages in flexible realizing three-dimensional micro-/nano-structures for a variety of materials (Liu et al., 2020; Fang et al., 2021; Somers et al., 2021; Wang et al., 2021).

In this minireview, we comprehensively reviewed recent progress on fabricating anti-icing/de-icing surfaces by femtosecond laser technologies. Typical bioinspired micro-/nano-structures fabrication strategies, superhydrophobic surfaces with anti-icing properties, and photothermal surfaces with de-icing properties are summarized. At last, we discussed challenges and prospects in anti-icing/de-icing surfaces fabricated by femtosecond laser technologies.

## 2 Fabrication of structured surfaces

**FIGURE 1:**
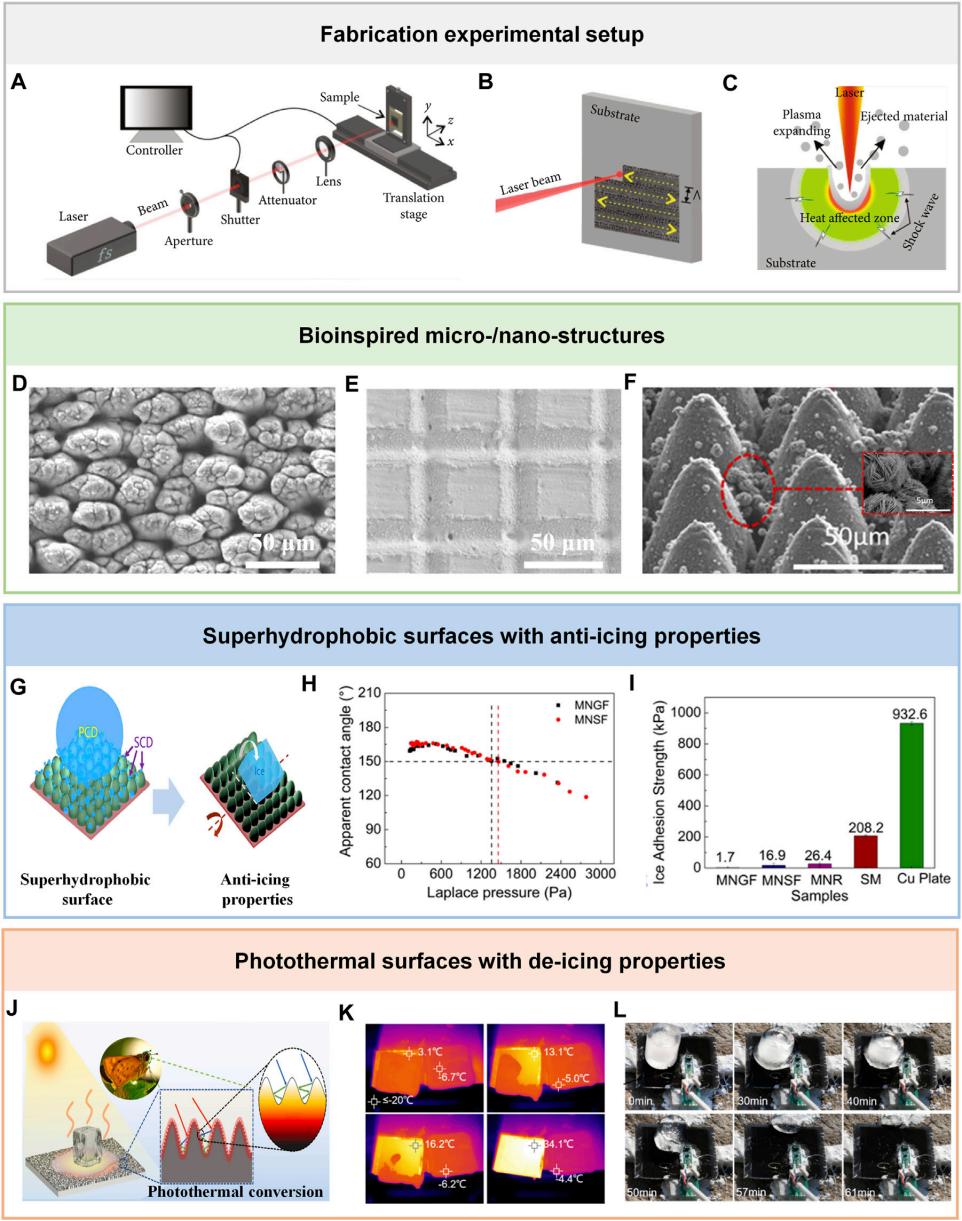
Fabrication of anti-icing/de-icing surfaces using the femtosecond laser. **(A)** Femtosecond laser direct writing experimental setup. **(B)** The scheme for laser scanning. **(C)** The scheme for the interaction between femtosecond laser and materials. Reproduced under the terms of the CC-BY Creative Commons Attribution 4.0 International License (Yong et al., 2022a) Copyright 2022, The Authors, Published by AAAS. Reproduced under the terms of the CC-BY Creative Commons Attribution 3.0 International License (Yong et al., 2022b) Copyright 2022, The Authors, Published by IOP Publishing Ltd. Reproduced from (Yong J. L. et al., 2019) with permission of American Chemical Society. **(D)** SEM images of mound structures. Reproduced from (Huang et al., 2018) with permission of Laser Institute of America. **(E)** SEM images of periodic square-shaped structures. Reproduced under the terms of the CC-BY Creative Commons Attribution 4.0 International License (Volpe et al., 2020) Copyright 2020, The Authors, Published by MDPI. **(F)** SEM images of triple-scale structures. Reproduced from (Pan et al., 2021) with permission of American Chemical Society. **(G)** The scheme for superhydrophobic surfaces with anti-icing properties. Reproduced from (Pan et al., 2021) with permission of American Chemical Society. **(H)** The relationship between CA and Laplace pressure. Reproduced from (Pan et al., 2021) with permission of American Chemical Society. **(I)** The ice adhesion strength of different superhydrophobic surfaces. Reproduced from (Pan et al., 2021) with permission of American Chemical Society. **(J)** The scheme for photothermal surfaces with de-icing properties. Reproduced from (Zhao et al., 2021) with permission of Elsevier. **(K)** The photothermal performance of laser-treated surfaces. Reproduced from (Chen et al., 2022) with permission of Elsevier. **(L)** Outdoor de-icing experiments. Reproduced from (Chen et al., 2022) with permission of Elsevier.

Micro-/nano-structures are essential in superhydrophobic anti-icing surface and photothermal de-icing surfaces. Figure 1A shows the scheme for typical laser processing equipment (Yong J. L. et al., 2019; Yong et al., 2022a; Yong et al., 2022b). A femtosecond laser is focused on the material surface through an objective lens. The sample is fixed on a translation stage. During the laser treatment process, femtosecond laser direct writing occurs on materials surfaces by moving the translation stage (Figures 1B,C). In the process of femtosecond laser treatment, high temperature and high pressure will be formed on the laser focus area (Zhang et al., 2017; Zhang and Sugioka, 2019; Zhang et al., 2020; Liu et al., 2022; Zhang D. S et al., 2022). Therefore, various bio-inspired structures have been fabricated for superhydrophobic anti-icing surfaces and photothermal de-icing surfaces.

Using the above-mentioned processing equipment, various bioinspired micro-/nano-structures, such as mound structures, periodic square-shaped structures, microcones structures, Siberian-cocklebur-like structures, and moth-eye-inspired structures, have been fabricated (Huang et al., 2018; Volpe et al., 2020; Ge et al., 2021; Pan et al., 2021). For example, as shown in Figure 1D, Huang et al. reported mound-structured surfaces on an aluminum alloy surface (Huang et al., 2018). The tall and short mound surfaces were fabricated by controlling the femtosecond laser fluence, laser spot radius, and different laser pulses. As shown in Figure 1E, Volpe et al. fabricated periodic square-shaped structures on aluminum alloy by scanning in parallel and perpendicular directions (Volpe et al., 2020). The depth is ∼8 μm, and the hatch distance is 10 μm–500 μm. Impressively, Pan et al. fabricated a triple-scale surface (Pan et al., 2021). The microcones were prepared by ultrafast laser ablation. The nano grasses and micro flower were formed on microcones structures after chemical oxidation (Figure 1F).

## 3 Superhydrophobic surfaces for anti-icing

Typically, the contact angle of water droplets on superhydrophobic surfaces is above 150°. Therefore, water droplets roll freely on superhydrophobic surfaces. The reason for superhydrophobic anti-icing surfaces are as follows (Figure 1G): i. Water droplets are hard to stay on superhydrophobic surfaces. ii. The formation time of ice crystals will be delayed. iii. The adhesion will be decreased.

When it comes to superhydrophobic surfaces fabricated by femtosecond laser technology, Huang et al. fabricated a superhydrophobic aluminum alloy surface by combining femtosecond laser technology with surface chemistry modification technology (Huang et al., 2018). The freezing delay can be up to 530 s because of excellent superhydrophobic properties. As a pioneer, Zhong’s group prepared a superhydrophobic surface with triple-scale structures (Pan et al., 2021). Notably, the contact angle of water drops is above 150° (Figure 1H). The ice adhesion is ∼1.7 kPa at -25°C (Figure 1I). Further, Zhong’s group developed superhydrophobic surfaces with robust icephobic performance by modification of polydimethylsiloxane on superhydrophobic surfaces (Che et al., 2022). In addition to post-modifying, Yin et al. prepared superhydrophobic polytetrafluoroethylene (PTFE) only by femtosecond laser technology (Yin et al., 2018). The contact angle of water drops is 157°. The water froze on the untreated PTFE after ∼14 min. In contrast, the water froze on the treated PTFE after ∼33 min.

## 4 Photothermal surfaces for de-icing

Photothermal surfaces convert solar energy into heat to melt the ice on the surface. Photothermal surfaces for de-icing show great features of low-cost and energy saving. As shown in Figure 1J, the incident light reflects between the micro-/nano-structures, reducing the reflectivity of materials and improving the absorption of materials. Therefore, sunlight is trapped in micro-/nano-structures, leading to enhancing light absorption.

It is worth noting that femtosecond laser technology can fabricate micro-/nano-structures to improve photothermal conversion ability for de-icing. For example, Zhao et al. fabricated moth-eye-inspired texturing surfaces for photothermal de-icing surfaces (Zhao et al., 2021). The remelted particles wrapped the micro-mountain, increasing optical path and light absorption. Moth-eye-inspired texturing surface temperatures rise from room temperature (∼30°C) to ∼80°C under one sun (1 kW/m^2^) irradiation for 300 s. After 180 s of illumination, the ice and melted water slide away. Moreover, Chen et al. prepared cauliflower-like surfaces for durable photothermal de-icing (Chen et al., 2022). Because of the combination of chemical reaction treatment, nanoscale structures are grown on the aluminum surface. The absorptivity reaches 97.3%. The high absorptivity results in better photothermal conversion capability, which is helpful to improve the photothermal deicing ability. The surface temperature increases by 48.5 °C within 300 s under one sun (1 kW/m^2^) irradiation (Figure 1K), and the ice can melt in 2 min (Figure 1L).

## 5 Conclusion and outlook

In this minireview, we comprehensively reviewed fabricating anti-icing/de-icing surfaces by femtosecond laser technologies. Typical bioinspired micro-/nano-structures fabrication strategies, superhydrophobic surfaces for anti-icing, and photothermal surfaces for de-icing are summarized. The superhydrophobic anti-icing surface and the photothermal de-icing surface depend on the bioinspired micro-/nano-structures. In the future, new concept micro-/nano-structures can be designed and fabricated to improve anti-icing and de-icing performance. For example, as a pioneer, Chen’s group reported the slippery liquid-infused porous surfaces for excellent ice resistance performance (Zhang J. L et al., 2022). The ice-delay time of slippery liquid infused porous surfaces was extended by 21.5% compared with the superhydrophobic surface. Furthermore, new fabrication technologies (such as laser interference and multi-beam parallel processing) will be explored to efficiently prepare large-area anti-icing and de-icing surfaces using an optical processing system design. In the future, significant progress will contribute to femtosecond laser technologies that enable anti-icing/de-icing surfaces into potential applications in aircraft, ships, and aerospace surfaces.
